# Fangchinoline, a Bisbenzylisoquinoline Alkaloid can Modulate Cytokine-Impelled Apoptosis via the Dual Regulation of NF-κB and AP-1 Pathways

**DOI:** 10.3390/molecules24173127

**Published:** 2019-08-28

**Authors:** Young Yun Jung, Muthu K. Shanmugam, Arunachalam Chinnathambi, Sulaiman Ali Alharbi, Omar H.M. Shair, Jae-Young Um, Gautam Sethi, Kwang Seok Ahn

**Affiliations:** 1 Department of Science in Korean Medicine, Kyung Hee University, 24 Kyungheedae-ro, Dongdaemun-gu, Seoul 02447, Korea; 2 Department of Pharmacology, Yong Loo Lin School of Medicine, National University of Singapore, Singapore 117600, Singapore; 3 Department of Botany and Microbiology, College of Science, King Saud University, Riyadh 11451, Saudi Arabia

**Keywords:** fangchinoline, NF-κB, AP-1, cancer, apoptosis

## Abstract

Fangchinoline (FCN) derived from Stephaniae tetrandrine S. Moore can be employed to treat fever, inflammation, rheumatism arthralgia, edema, dysuria, athlete’s foot, and swollen wet sores. FCN can exhibit a plethora of anti-neoplastic effects although its precise mode of action still remains to be deciphered. Nuclear factor-κB (NF-κB) and activator protein-1 (AP-1) can closely regulate carcinogenesis and thus we analyzed the possible action of FCN may have on these two signaling cascades in tumor cells. The effect of FCN on NF-κB and AP-1 signaling cascades and its downstream functions was deciphered using diverse assays in both human chronic myeloid leukemia (KBM5) and multiple myeloma (U266). FCN attenuated growth of both leukemic and multiple myeloma cells and repressed NF-κB, and AP-1 activation through diverse mechanisms, including attenuation of phosphorylation of IκB kinase (IKK) and p65. Furthermore, FCN could also cause significant enhancement in TNFα-driven apoptosis as studied by various molecular techniques. Thus, FCN may exhibit potent anti-neoplastic effects by affecting diverse oncogenic pathways and may be employed as pro-apoptotic agent against various malignancies.

## 1. Introduction

Bioactive natural product compounds have been used in traditional medical practices especially in the Asian continent for centuries [1,2,3,4,5,6,7,8,9,10,11,12,13,14,15,16,17,18,19]. Recent advances in research and development have identified several novel molecules with potent bioactivity in several models of cancer [4,20,21,22,23,24,25,26,27,28]. Cancer especially in advanced stages is still considered incurable [29,30] and several pharmacological agents designated for cancer therapy can be obtained from Mother nature [21]. Leukemia comprises a group of hematological malignancies and is characterized by abnormal white cells that originate from the bone marrow and can affect both adults and children [31,32]. Despite remarkable progress in leukemia treatment, overall progress still remains riddled with frequent relapses and high mortality [33]. 

Hyperactivation of nuclear factor-κB (NF-κB) and activator protein-1 (AP-1) is commonly seen in varying chronic conditions including cancers [13,14,31,34,35,36,37,38,39,40,41,42]. NF-κB can be frequently activated in both solid tumors as well as in those of cells of blood-forming tissues [43,44,45,46,47,48,49,50,51,52,53,54,55,56]. NF-κB may be activated by carcinogens, pro-oxidants, and growth factors and drives the oncogenic process [49,55,56,57,58,59,60,61,62,63,64]. Tumor necrosis factor-alpha (TNFα), was initially reported to be a potent anti-tumor cytokine that can abrogate tumor growth [65,66,67,68,69]. However, it was later found that TNFα can also function as a potent cytokine that is capable of inducing NF-κB signaling cascade and mediate tumorigenesis [65,70]. 

NF-κB, a complex of p50, p65, and IκBα, stays in the cytoplasm under unstimulated conditions [44,45,46,47,58,71]. Upon activation, several upstream kinases, such as IκB kinase (IKK), are phosphorylated and can ubiquitinate and degrade IκBα to release NF-κB which subsequently moves inside the nucleus and regulates transcription of genes controlling, apoptosis, angiogenesis, metastasis, and chemo- and radio-resistance [44,45,46,52,57,59,65,72,73]. Similarly, AP-1 has been reported to be overexpressed in different neoplastic cells and can also closely regulate the hallmarks of carcinogenesis [37,74,75]. Several AP-1 target genes, such as matrix metalloproteinases [MMPs] (MMP-1, MMP-3 and MMP-9), extracellular matrix associated proteins, and protein kinase C, have been implicated in carcinogenesis [76,77,78]. Furthermore, AP-1 has also been found to interact with NF-κB and augment cancer progression [38,79,80]. Therefore, dual targeting the NF-κB/AP-1 signaling axis may be effective for cancer therapy. 

Fangchinoline (FCN) is a bisbenzylisoquinoline that belongs to the family Menispermaceae [10,31,81]. It can display significant anti-tumoral effects against malignant cells and in breast cancer cells, FCN inhibited cell proliferation and induced cell death that was driven by the mitochondrial pathway [82,83,84]. Similarly, FCN was observed to suppress the phosphorylation of AKT (phospho-Thr308), cyclin D1, and MMP-2, MMP-9 levels, as well as upregulate caspase-3 and -8 in MG63 and U2OS osteosarcoma cells [85]. In another report by Guo et al., FCN was noted to repress invasion by suppression of FAK-AKT and FAK-MEK-ERK1/2 pathways [86]. It has also been documented to mitigate metastasis of melanoma cells by modulating focal adhesion kinase (FAK) phosphorylation [87]. 

In grade IV human glioblastoma multiforme cells (U87 MG and U118 MG), FCN interfered with AKT signaling pathway and caused apoptosis [88]. In another study, FCN negatively affected the multiplication in prostate cancer cells by suppressing the articulation of genes controlling cell-cycle regulation [89]. In SPC-A-1 lung adenocarcinoma cells, it also abrogated cellular growth and caused apoptosis [90]. Chronic myeloid leukemia is predominantly characterized by BCR-ABL tyrosine kinase deregulation. FCN can also affect the proliferation of K562 cells by causing arrest at G0/G1 phase, upregulating CDKN1A and MCL1 mRNA with concomitant downregulation of cyclin D1 mRNA [91]. Interestingly, FCN displayed autophagy mediated apoptosis in bladder cancer cells and caused a reduction in cellular ATP levels, upregulated LC3-II/LC3-1 ratio and caspase-3 and down regulated p62 protein levels [92]. In another study by Wang et al., it was found to promote autophagic cell death by modulating the p53/sestrin 2/AMPK signaling in human hepatocellular carcinoma cells [93]. Interestingly, FCN could also inhibit pancreatic cancer cell growth via modulating NR4A1 dependent apoptotic pathway [94]. 

In this study, we primarily deciphered the action of FCN in regulating NF-κB/AP-1 cell signaling and survival pathways and aimed to understand the effect of this alkaloid in regulating cytokine-induced apoptosis.

## 2. Results

### 2.1. FCN Abrogates NF-κB/AP-1 Activation

**Figure 1:**
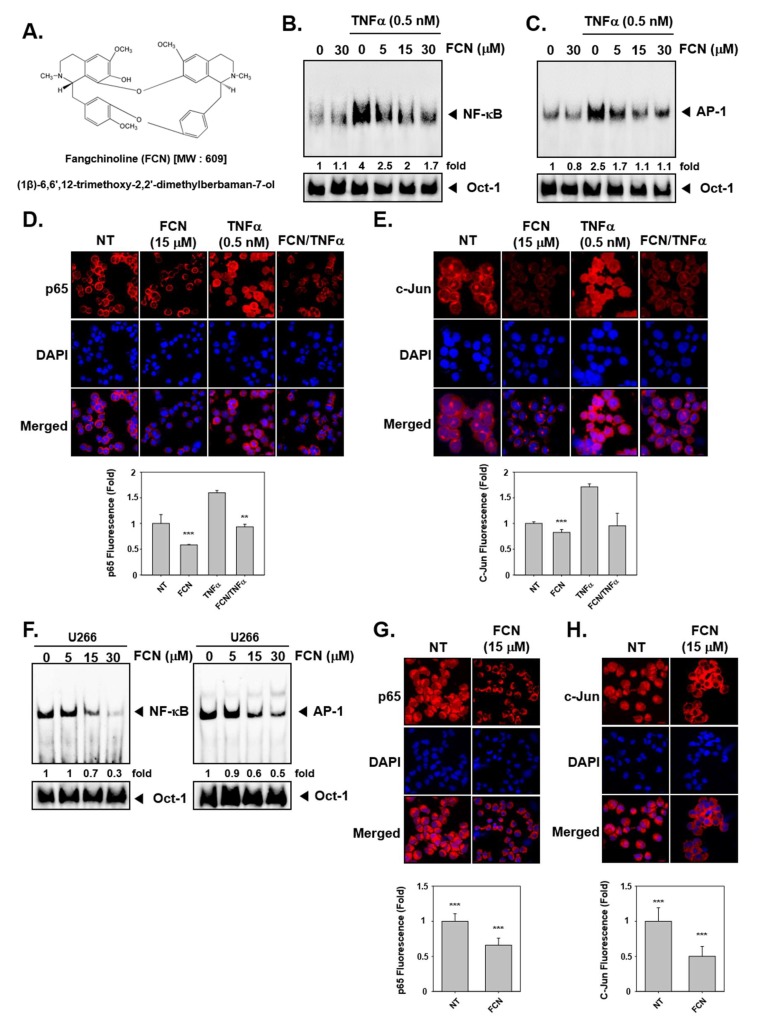
Fangchinoline (FCN) affects the activation of oncogenic transcription factors. (**A**) The chemical structure of FCN. (**B**,**C**) FCN was added in different concentrations to KBM5 cells. After 2 h of treatment, 0.5 nM of TNFα was added for 15 min. Nuclear extracts (NE) were prepared for electrophoretic mobility shift assay (EMSA) assay to evaluate Nuclear factor-κB (NF-κB) and activator protein-1 (AP-1) expression. Oct-1 was used as loading control. (**D**,**E**) KBM5 cells were treated as described above. Immunocytochemistry was done to analyze p65 and c-Jun translocation to the nucleus, results were compared between non-treated (NT) and FCN treated cells. (**F**) Changes in constitutive NF-κB and AP-1 expression upon FCN treatment in U266 cells was evaluated by EMSA assay. Oct-1 was used as loading control. (**G**,**H**) p65 and c-Jun nuclear translocation was evaluated by immunocytochemistry in U266 cells. The results shown are representative of at least three independent experiments.

To evaluate whether FCN can regulate TNFα-induced NF-κB/AP-1 activation in KBM5 cells, we performed an electrophoretic mobility shift assay (EMSA) assay. We had previously measured cytotoxicity with of FCN in KBM5 cells and found that IC_50_ (half-maximal inhibitory concentration) was 10 μM at 72 h. So we selected the concentration in the range 0 to 30 μM, around the IC_50_ value for duration up to 24 h and no significant toxicity was noted in this dose range. The results indicated that NF-κB/AP-1 proteins can be activated by TNFα (0.5 nM) exposure and suppressed by FCN treatment in concentration response studies (Figure 1B,C). In addition, in immunocytochemistry results, nuclear translocation of p65 and c-Jun was induced by TNFα and FCN suppressed this activity (Figure 1D,E). Interestingly, similar effects on DNA binding and nuclear translocation was observed in myeloma U266 cells (Figure 1F–H).

### 2.2. FCN Mitigates TNFα -Induced IKK Phosphorylation

**Figure 2:**
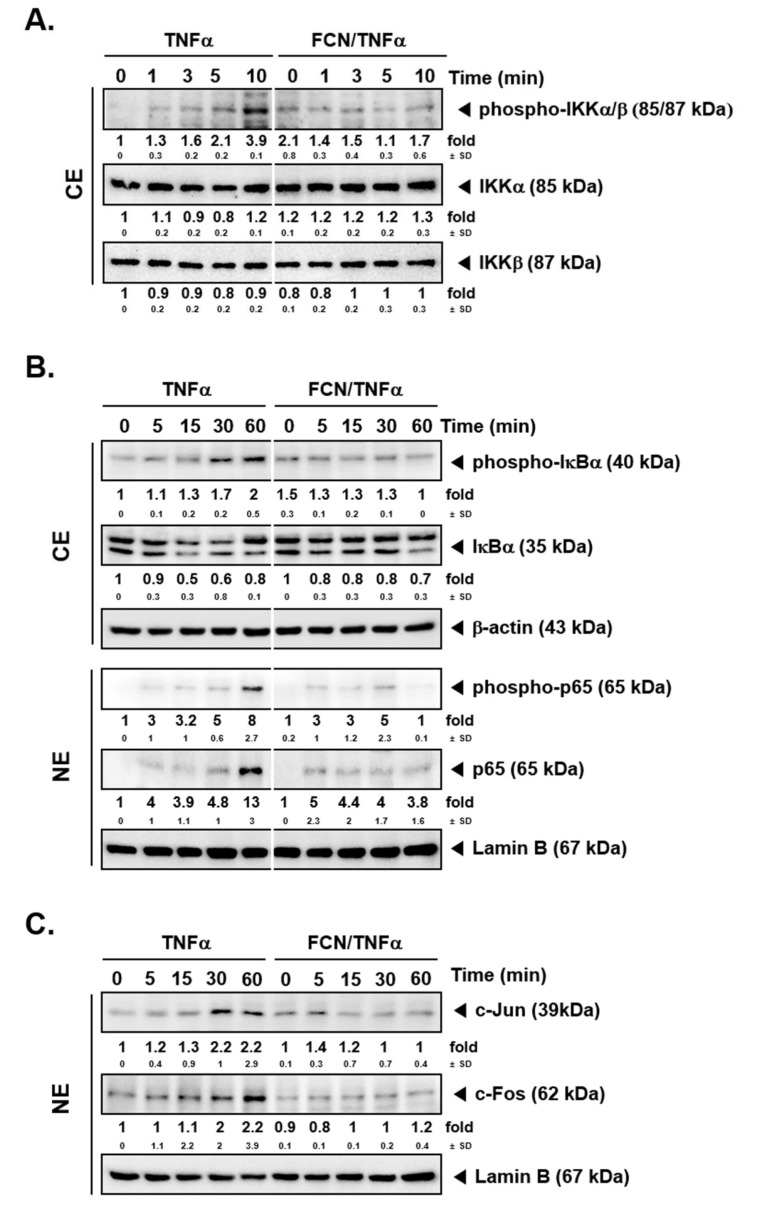
FCN affects NF-κB activation and IκBα degradation in KBM5 cells. KBM5 cells were pre-treated with 15 μM of FCN for 2 h. After pre-treatment, 0.5 nM TNFα was added for the indicated time intervals. (**A**) Cytoplasmic extracts (CE) were prepared to evaluate the expression of phospho-IKKα/β, IKKα, and IKKβ by Western blot analysis. (**B**) Cytoplasmic extracts (CE) and nuclear extracts (NE) were analyzed to evaluate NF-κB activation and IκBα degradation. (**C**) AP-1 related c-Jun and c-Fos expression was determined by Western blot analysis using nuclear extracts (NE). The results shown are representative of at least three independent experiments.

To determine whether FCN has modulatory effects on IKK activation, FCN (15 μM) pre-treated KBM5 cells were first stimulated with TNFα. Western blot experiments carried out thereafter suggest that FCN suppressed phospho-IKKα/β activation (Figure 2A). Because IκBα degradation can lead to NF-κB nuclear translocation activation, we evaluated whether FCN can also inhibit IκBα degradation. As shown, FCN can effectively abrogate IκBα degradation as well as phosphorylation of p65 driven by TNFα exposure (Figure 2B), and it also attenuated the expression of c-Jun and c-Fos proteins (Figure 2C).

### 2.3. FCN down Regulates Levels of Diverse Oncogenic Proteins

**Figure 3:**
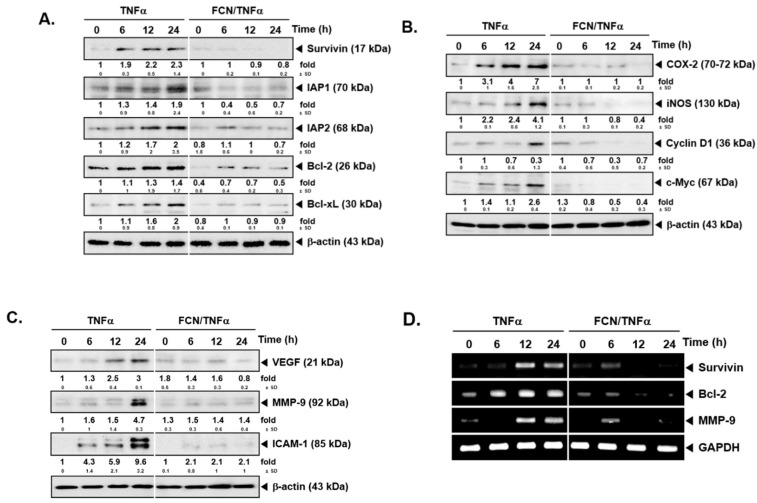
FCN influences the expression of various oncogenic genes. KBM5 cells were pre-treated with 15 μM of FCN for 2 h. After 2 h, 0.5 nM TNFα was added for 24 h. (**A**) Western blot analysis was performed to check the expression of various proteins. (**B**) The levels of proliferative gene products were analyzed by Western blot analysis. (**C**) The expression of metastatic gene products were analyzed by Western blot analysis. (**D**) RT-PCR was done to check the mRNA level of various genes. The results shown are representative of at least three independent experiments.

To analyze whether FCN has potential effects on the levels of various gene products, we performed Western blot analysis and RT-PCR. Among various gene products, first, we evaluated the effect of FCN on survivin, IAP-1, IAP2, Bcl-2, and Bcl-xl proteins. As shown on Figure 3A, TNFα caused an increase but FCN treatment downmodulated the expression of these proteins. Additionally, we found that FCN treatment could mitigate COX-2, Cyclin D1, and c-Myc expression (Figure 3B). Moreover, tumor cell metastasis related gene products, such as VEGF, MMP-9, and ICAM-1, could be mitigated by FCN (Figure 3C). Furthermore, we have selected few significant markers, such as survivin, Bcl-2, and MMP-9, for RT-PCR analysis that may represent important hallmarks of cancer [95], and the appearance of survivin, Bcl-2, and MMP-9 genes were substantially repressed upon FCN exposure (Figure 3D).

### 2.4. FCN Enhances TNFα-Induced Apoptosis through Affecting Caspase-3 Activation

**Figure 4:**
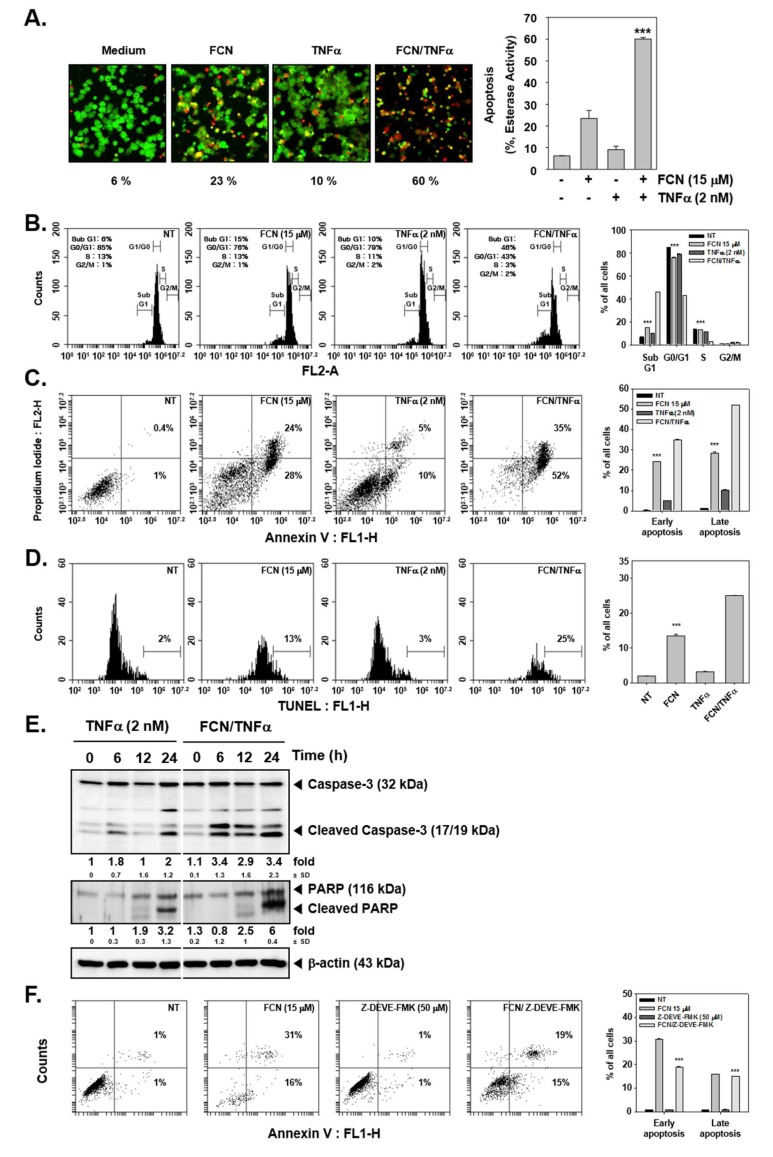
FCN can augment cytokine caused apoptosis. KBM5 cells were pre-treated with 15 μM of FCN and 2 nM TNFα was thereafter added for a total of 24 h. (**A**) Cytotoxicity of FCN and TNFα was analyzed by live and dead assay. Live cells were stained with Calcein AM (green) and dead cells were stained with Ethd-1 (red). The graph (*right)* shows the rate of dead cells by quantification. (**B**) Cells were treated as described above and then fixed with EtOH overnight. RNase A (10 μg/mL) was treated for 1 h and then cells were stained with propidium iodide, analyzed by flow cytometry. (**C**) FCN and TNFα treated cells were stained with Annexin V Fluorescein isothiocyanate (FITC)/propidium iodide, and analyzed by flow cytometry. (**D**) After FCN and TNFα treatment, cells were subjected to Terminal deoxynucleotidyl transferase dUTP nick end labeling (TUNEL) assay. (**E**) KBM5 cells were treated as described above and Western blot analysis was performed. (**F**) KBM5 cell were treated with FCN (15 μM) and Z-Asp(O-Me)-Glu(O-Me)-Val-Asp(O-Me) fluoromethyl ketone (Z-DEVD-FMK) (50 μM, caspase inhibitor) for 24 h. Cells were stained with Annexin V FITC/propidium iodide and then analyzed by flow cytometry. The results shown are representative at least three independent experiments.

We first evaluated whether TNFα-induced apoptosis may be augmented upon FCN exposure by live and dead assay, cell cycle analysis, annexin V, and TUNEL assays. First, FCN and TNFα treated cells were probed with calcein AM and Ethd-1. Because live cells can disaggregate the calcein, cells appeared in green color. On the contrary, dead cells exhibited damage in their cell membranes, so Ethd-1 can invade into the cells through ruptured membranes and the cells thus appeared as red colored (Figure 4A). Moreover, it was observed that FCN exposure increased sub G1 phase from 6% to 15%, TNFα increased sub G1 phase from 6% to 10%, and combination treatment clearly enhanced distribution in sub G1 phase to 46% (Figure 4B). 

Interestingly in annexin V assay, FCN treatment could increase both early and late apoptosis form 0.4% and 1% to 24% and 28%. TNFα also exacerbated early and late apoptosis to 5% and 10%. Interestingly, combination treatment enhanced apoptosis to 35% and 52% (Figure 4C). In addition, we analyzed the effect on apoptosis by TUNEL assay. We noted that FCN induced apoptosis from 2% to 13% and combination treatment prominently enhanced apoptosis to 25% (Figure 4D). We later examined the mechanism(s) behind enhancement of apoptosis observed upon FCN treatment. As shown in Figure 4E, TNFα treatment induced apoptosis and FCN exposure clearly augmented cell death through augmentation in caspase-3 as well as PARP cleavage. We additionally confirmed that FCN indeed caused apoptosis through caspase cleavage. KBM5 cells were treated with Z-DEVD-FMK (50 μM), known as caspase inhibitor, and FCN (15 μM) and as illustrated in Figure 4F, FCN can induce substantial apoptosis but Z-DEVD-FMK treatment could attenuate apoptosis. Overall, the results demonstrated that FCN induced apoptosis through the caspase and PARP dependent pathways.

**Figure 5:**
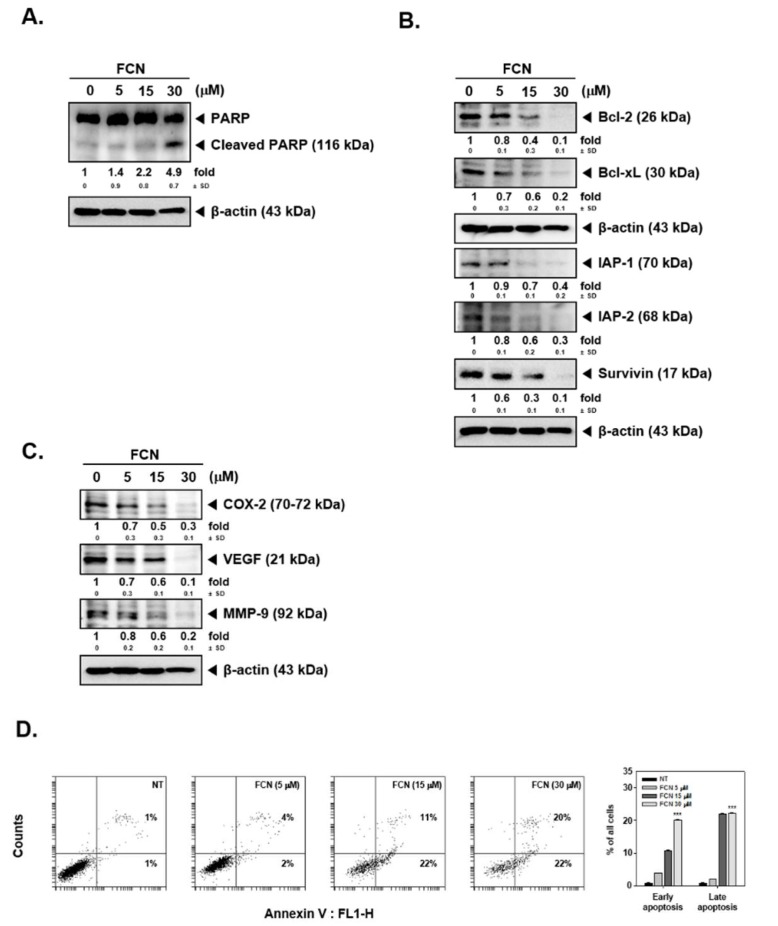
FCN induced apoptosis in U266 cells. U266 cells were treated with FCN (0, 5, 15, and 30 μM) for 24 h. Whole cell lysates were prepared to analyze the expression of (**A**) PARP and (**B**) various oncogenic gene products such as Bcl-2, Bcl-xl, IAP-1, IAP-2, survivin, as well as (**C**) COX-2, VEGF, and MMP-9. (**D**) FCN-induced apoptosis in U266 cells was analyzed by annexin V assay. Cells were treated with FCN for 24 h. The results shown are representative at least three independent experiments.

Moreover, as shown in Figure 5A,B, FCN treatment induced PARP cleavage and attenuated the level of diverse oncogenic proteins in myeloma cells as well. Furthermore, cell death in these cells increased dramatically with increasing concentrations of FCN (0, 5, 15, 30 μM), (Figure 5C). 

## 3. Discussion

**Figure 6:**
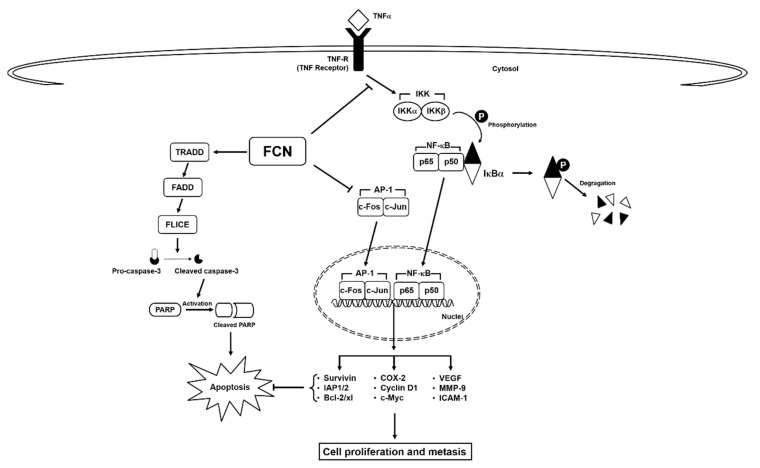
A schematic diagram of FCN effects on NF-κB/AP-1 in tumor cells.

Here, we deciphered the anti-neoplastic actions of FCN in abrogating the survival of chronic myeloid leukemia cells. FCN is a bisbenzylisoquinoline based alkaloid that has been documented to act as a potent anti-neoplastic agent against different malignancies [10]. Leukemia, a cancer characterized by abnormal growth of blood cells [96], can exist in various forms, such as acute lymphocytic leukemia, acute myeloid leukemia, chronic lymphocytic leukemia, and chronic myeloid leukemia [32,79,97,98,99,100]. NF-κB has been known to play an important role in the regulation of cell survival, proliferation, and metastasis [101]. It is also well known that cytokine TNFα can regulate the robust activation of master transcription factor NF-κB [65,70], and persistent NF-κB overexpression/phosphorylation has been detected in leukemia as well as multiple myeloma [54,102,103,104,105]. FCN has been found to inhibit the growth of chronic myeloid leukemia K562 cells, but detailed mode of its anti-cancer actions still remains unclear [91]. Thus, in this study we employed both human chronic myeloid leukemia and myeloma cell lines to decipher the primary mode(s) of action regulating the anti-neoplastic actions of FCN. We noted that FCN effectively suppressed both constitutive and induced NF-κB and AP-1 activation as well as modulated the survival potential of the tumor cells (Figure 6). 

Interestingly, we found that FCN-induced NF-κB may be caused by the abrogation of inhibitor kappa kinase (IKK) activation and suppression of IκBα phosphorylation. These steps are important for the transcription of myriad of genes controlled by NF-κB [14,44,79,100]. Next, FCN was observed to mitigate nuclear localization of p65 and effectively promote a down-modulation in the levels of pro-survival as well as oncogenic proteins. As known, translocation of NF-κB complexes into the nucleus is an essential step for its reported oncogenic functions [43,44]. Interestingly, in a previous study, FCN was noted to promote cell death and suppress migration via regulation of NF-κB activation in mammary tumor cells [22]. The AP-1 complex can drive oncogenesis in different malignancies including leukemia and myeloma [13,106]. Uncontrolled cellular proliferation has been correlated to activation of c-fos and c-jun proteins and inadequate response to chemotherapeutic agents [14,38]. Moreover, c-fos and c-jun can be overexpressed, and mediate process of oncogenic transformation in leukemic cells, however, normal lymphocytes did not express c-Jun [32,75,80]. However, whether FCN can affect NF-κB and AP-1 activation by modulating the phosphorylation of an upstream signaling molecule that may regulate both these signaling cascades requires additional experiments.

It is understood that simultaneously attenuating both NF-κB and AP-1 pathways can be an important approach to target oncogenesis [1,107,108]. FCN could significantly enhance cytokine-induced apoptosis and this effect was found to be mediated via its action in inducing capase-3 activation and subsequent PARP cleavage. Thus, our finding suggests that NF-κB and AP-1 may augment pro-survival signaling mechanism(s) in malignant cells and therapeutic targeting of these two potent transcription factors by FCN could abrogate tumor growth as well as survival (Figure 5). Overall, it appears that FCN can act as a promising anti-cancer drug whose potential remains to be validated in appropriate tumor models. In addition, further studies are needed to determine whether FCN can be employed along with existing treatment modalities for cancer therapy.

## 4. Materials and Methods

### 4.1. Reagents

Fangchinoline (FCN, Figure 1A) was purchased from Chem faces (Wuhan, Hubei, China). FCN was stored in 100 mM stock solution with dimethyl sulfoxide at −20 °C and diluted in cultured media for in vitro experiments. LightShift^®^ Chemiluminescent EMSA kit was purchased from Thermo Fisher Scientific Inc. Alexa Fluor^®^ 594 donkey anti-rabbit IgG (H + L) antibody was obtained from Life Technologies (Grand Island, NY, USA). Z-DEVD-FMK (caspase-3 inhibitor) was purchased from CALBIOCHEM (San Diego, CA, USA).

### 4.2. Cell Lines and Culture Conditions

Human chronic myeloid leukemia (KBM5) cells as described before [109] were cultured in IMDM medium containing 10% inactivated fetal bovine serum (FBS) and 1% penicillin-streptomycin. Human multiple myeloma (U266) cells as described before [54] were cultured with RPMI medium containing 10% fetal bovine serum (FBS) and 1% penicillin-streptomycin. 

### 4.3. Electrophoretic Mobility Shift Assay (EMSA)

NF-κB and AP-1 DNA binding were analyzed by EMSA as reported before [38,109].

### 4.4. Western Blot Analysis 

Western blot assay was done as explained before [110,111].

### 4.5. RT-PCR

RT-PCR was carried out as elaborated before [112].

### 4.6. Immunocytochemistry

Immunohistochemistry was done as described previously [109].

### 4.7. Cell Cycle Analysis

To evaluate apoptotic effects of FCN, cells were pre-treated with FCN (15 μM) for 2 h and TNFα (2 nM) treated for total 24 h. After treatment, cells were washed by 1× PBS and fixed with 100% EtOH for overnight at 4 °C. Cells were resuspended with fresh 1× PBS as well as RNase A (1 μg/mL) treated at 37 °C for 1 h and then stained with propidium iodide. Thereafter analysis was carried out by BD Accuri™ C6 Plus Flow Cytometer (BD Biosciences, Becton-Dickinson, Franklin Lakes, NJ, USA). 

### 4.8. TUNEL Assay

Annexin V assay was done as explained before [113].

### 4.9. Live and Dead Assay

KBM5 cells were pre-treated with FCN (15 μM) and TNFα (2 nM) for total 24 h. After treatment, cells were incubated with 5 μM Calcein AM and 5 μM Ethd-1(Ethidium homodimer-1) at 37 °C for 30 min. Thereafter Olympus FluoView FV1000 confocal microscope was used for analysis (Tokyo, Japan).

### 4.10. Statistical Analysis

Statistical significance was calculated by Mann–Whitney U test. Significance was set at *p* < 0.05.

## Abbreviations

NF-κB	nuclear factor kappa-light-chain-enhancer of activated B cells
AP-1	activator protein 1
TNFα	tumor necrosis factor alpha
IKKs	inhibitory kappa B kinases
IκBα	nuclear factor of kappa light polypeptide gene enhancer in B cells inhibitor alpha
COX-2	cyclooxygenase-2
MMP	mitochondrial membrane potential
PARP	poly (ADP-ribose) polymerase
IC_50_	half maximal inhibitory concentration
